# Elementary Observations: Building Blocks of Physical Information Gain

**DOI:** 10.3390/e26080619

**Published:** 2024-07-23

**Authors:** J. Gerhard Müller

**Affiliations:** Department of Applied Sciences and Mechatronics, Munich University of Applied Sciences, D-80335 Munich, Germany; gerhard.mueller@hm.edu or jgmuegra@t-online.de

**Keywords:** physical measurement, information gain, event generation, physical action, energy dissipation, spacetime extension, Landauer principle

## Abstract

In this paper, we are concerned with the process of experimental information gain. Building on previous work, we show that this is a discontinuous process in which the initiating quantum-mechanical matter–instrument interactions are being turned into macroscopically observable events (EOs). In the course of time, such EOs evolve into spatio-temporal patterns of EOs, which allow conceivable alternatives of physical explanation to be distinguished. Focusing on the specific case of photon detection, we show that during their lifetimes, EOs proceed through the four phases of initiation, detection, erasure and reset. Once generated, the observational value of EOs can be measured in units of the Planck quantum of physical action $h=4.136\times 10^{-15}eVs$. Once terminated, each unit of entropy of size $k_{B}=8.617\times 10^{-5}eV/K$, which had been created in the instrument during the observational phase, needs to be removed from the instrument to ready it for a new round of photon detection. This withdrawal of entropy takes place at an energetic cost of at least two units of the Landauer minimum energy bound of $E_{La}=\ln\left(2\right)k_{B}T_{D}$ for each unit of entropy of size $k_{B}$.

## 1. Introduction

The idea that matter is composed of indivisible elementary building blocks has been around since ancient times and has become widely known under the headline of “Greek atomism [1]”. The mental concept of atomism turned into a seriously considered scientific reality with the rise of modern chemistry and the discovery of the periodic table of elements [2]. At its time of publication, the atoms in the periodic table were still considered indivisible building blocks of matter, a point of view that was shattered in the early years of the 20th century with the discovery of the electron [3], the proton [4], and later with the neutron [5]. In the year of 1932, when the neutron was discovered, the idea emerged that all matter in the universe might ultimately be composed of only three kinds of elementary particles. This idea was later shattered by the research into cosmic rays, and even more by experiments with high-energy accelerators [6]. The bewildering variety of “elementary particles” that emerged from this research was later consolidated into the standard model of elementary particles [6], which contains no less than 18 constituent particles. The increasing number of “elementary particles” raised an increasing discomfort in the scientific community, a situation that has been aggravated as new ideas of unexplained “dark matter“ and “dark energy” have come up [7,8].

Returning to the subject matter of this paper, it is suggested that ultimate simplicity may not necessarily exist on the level of matter but rather on the level of observation. Being inspired by the idea of John Archibald Wheeler [9] that all physical entities at their bottom might be information-theoretic in origin, we have re-considered in a recent paper [10] three key experiments that were groundbreaking in the evolution of our modern ideas of matter at the atomic, nuclear and elementary particle scales, with an informational perspective in mind. The experiments re-considered were the Rutherford scattering experiments of Geiger and Marsden [11,12], the double-slit experiments with photons, electrons and other pieces of matter [13,14,15,16], and the visualization of nuclear particle trajectories in cloud, bubble and streaming chambers [17,18,19]. In re-considering these key experiments, we have taken the standpoint that these experiments can be regarded as questions posed to nature, and we asked ourselves how these questions are being answered by nature itself. A key result was that in spite of the different questions posed, all experimental answers are structured in a remarkably similar way, namely, in producing streams of macroscopically observable events (EOs) in the first place, which accumulate in the long run into spatio-temporal patterns of EOs, which represent the expected experimental answers, and which finally allow decisions to be taken regarding the validity of competing alternatives of physical explanation. In this previous work [10], we also arrived at the conclusion that EOs by themselves exhibit a double nature, being both abstract pieces of binary information, on the one hand, and concrete physical entities, endowed with the property of physical action, on the other hand:

(1) $$W_{obs}=E_{obs}\tau_{obs}\gg h.$$

In this equation, $E_{obs}$ stands for the energy expended in producing a macroscopically observable EO, $\tau_{obs}$ for the lifetime during which the EO had remained macroscopically observable and h for Planck’s constant of physical action. The inequality $W_{obs}\gg h$, moreover, indicates that EOs can be regarded as macroscopic images of the initiating micro-events and that the observational value of EOs can be measured in multiples of the elementary quantum of h. In this paper, we concentrate on EOs that have been produced by photon–matter interactions, and we aim to arrive at a set of figures of merit (FOMs) that characterize the observational value and the statistical significance of the produced EOs. Finally, we assess the energetic and entropic costs of producing such EOs.

## 2. Photons as Key Carriers of Physical Information

**Figure 1:**
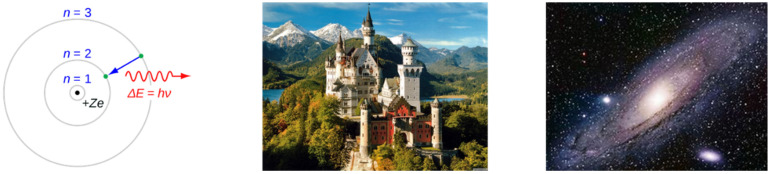
Situations where matter becomes visible through the interaction with photons.

The choice of concentrating on photons and the process of photon detection is motivated by the fact that photons are the most important carriers of information, and that photon detection is the key process through which our material world becomes visible and accessible to physical investigation. This latter fact is demonstrated in a cartoon-like manner in Figure 1.

The above figure demonstrates that the information-carrying potential of photons is not at all limited to normal macroscopic dimensions, but that it extends all throughout the entire range of atomic and nuclear dimensions up to the length scale of truly cosmic dimensions measuring in billions of light years. We show in a mathematical appendix (Appendix A) that this information-carrying capacity of photons derives from the fact that in the course of electromagnetic wave propagation, quanta of physical action of size h are continually generated and erased while being shifted in space in discrete steps of length Δx=λ/2, where λ is the photon wavelength and x the direction of propagation. Such propagation processes continue up to the point at which the quanta of physical action become absorbed either inside a passive piece of matter or in some kind of photon detector. Whereas in both cases the quanta of physical action are absorbed, a macroscopically observable output signal is generated when the absorption takes place inside a suitably designed photon detector. The above figure, moreover, shows that without the possibility of photon detection, we would not be able to observe our own macroscopic environments, nor would the sciences of physics and astronomy exist at all, as without electromagnetic interaction and the possibility of photon detection, all kinds of matter would disappear into eternal darkness and unobservability.

**Figure 2:**
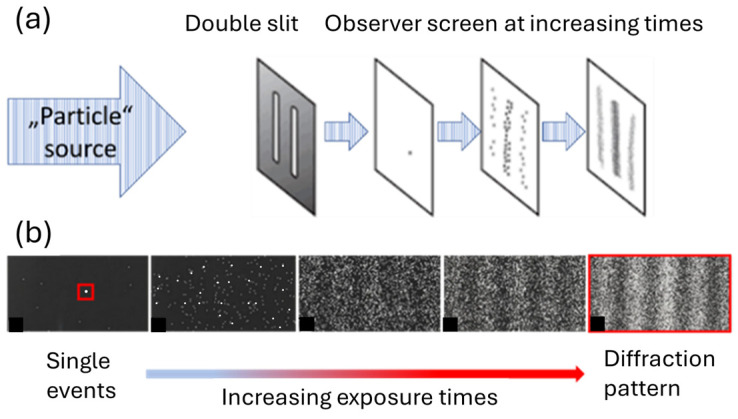
(**a**) Sketch of a double-slit experiment with photons, conducted for increasingly longer periods of time. Photon impacts on the detector screen feature as black dots; (**b**) developed photographic plates exposed to photons for increasingly longer times. After development of the photographic plates, individual “photon impacts” appear as small, permanently whitened spots, approximating diffraction patterns in the long run.

In Figure 2, we also sketch in a cartoon-like manner an experiment in which single photons were processed in a one-by-one manner through a double-slit arrangement, fitted with a fluorescent detection screen behind the double slits [13]. This picture vividly demonstrates that each single photon becomes detected in the form of a “particle impact” and that the individual “particle impacts” converge in the long run towards a “diffraction pattern”, which is taken as evidence that photons, on their travel from source to detection screen, propagate in the form of waves that had become diffracted upon passage through the double-slit arrangement.

Looking at this same experiment from an informational point of view, it is suggested that the individual “particle impacts” are binary pieces of information that decide between two simple alternatives (event has happened at the particular “impact site”/event has not happened at this “site”). As the decision of a binary alternative provides the minimum possible information gain of one single binary digit, the seeming “particle impacts” can rightly be considered as “elementary observations (EOs)” and the “diffraction patterns” that emerge in the long run as complex pieces of information, built up from individual EOs. This latter interpretation puts well-designed physical experiments in parallel with technical information channels in which meaningful messages, such as texts and images, are transported from information source to information sink in the form of meaningless binary bits [20,21,22,23].

As similar conclusions concerning EOs were also reached in our previous work [10] with regard to the individual light flashes produced in a Rutherford scattering experiment, or the individual liquid droplets that had formed around ionized particle tracks in a cloud chamber, we extrapolated that experimental answers, in general, are being built up in a discontinuous manner from elementary pieces of information, i.e., EOs. Once looked at as wholes, such experimental answers raise informational questions on two different levels:

(a) What is the physical and informational nature of the individual EOs?
(b) What are the mental processes that allow physical meaning to be assigned to the patterns of EOs that emerge from the accumulation of huge numbers of EOs?

In the present paper, we concentrate on the first question and leave the discussion of the second question to a forthcoming paper [24].

## 3. Photon Detection with the Help of a Conceptual Device

**Figure 3:**
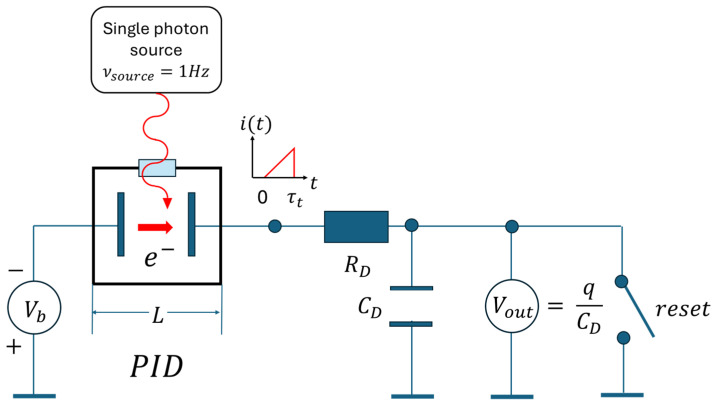
Schematic view onto a photo-ionization detector (PID). While the thick red arrow inside the box indicates the internal photoelectron current flow, the thin blue lines on the exterior are electrical wires that allow for current continuity throughout the whole device; $R_{D}$ and $C_{D}$ form an integrator circuit that converts the very short electron pulses into quasi-permanent output voltage readings. The frequency $\nu_{source}$ that is much lower than the inverse transit time through the electrode gap was chosen to conform with the conditions of single-photon detection.

In the following, we present the idea of a conceptual device that turns quantum-mechanical photon–matter interactions into macroscopically observable EOs. The principal architecture of such a device is sketched in Figure 3. There, a photo-ionization detector (PID) is shown, which consists of a pair of metal or semiconductor electrodes that are positioned facing each other in the form of a parallel-plate capacitor that is housed in a fully evacuated box with side lengths L and an entrance window through which photons can penetrate this box. In case an externally generated photon gets trapped inside this box, and in case the energy $E_{ph}$ of this photon exceeds the electron work function $q\phi_{m}$ of the metal or semiconductor electrodes, a mobile electron is generated that in principle can flow from one electrode to the other. Provided a bias potential, $V_{b}$, is applied across the electrode pair, a directional electron current is initiated, which takes the form of a triangular current pulse, and which can be observed in the external circuit. Inside this circuit, a macroscopically observable image of the initiating micro-event of photon excitation is formed. While the generation of the mobile electron is an intrinsically quantum-mechanical measurement process that likely proceeds through the process of wavefunction collapse, the transport of the liberated electron is a process that can be described in a purely classical manner, and which thus allows those processes to be elucidated in which an intrinsically unobservable micro-event is transformed into a macroscopically observable event, i.e., into an EO.

In the present paper we build on the results obtained in our first paper on photon detection [25] where we have shown that the technical performance parameters [26] of signal-to-noise ratio (SN), noise-equivalent power (NEP) and detectivity (D and $D^{*}$) can be neatly translated into informational language by making the following assignment:

(2) $$i_{D}\left(E_{ph},T_{D}\right)=\frac{1}{ln(2)}ln\left[SN\right]=\frac{1}{ln(2)}ln\left[\frac{N_{s}}{N_{n}}\right]$$

There, SN is the conventionally defined signal-to-noise ratio, $N_{s}$ and $N_{n}$ the numbers of signal and noise electrons that build up the output signal currents $I_{s}\left(t\right)$ and $i_{D}\left(E_{ph},T_{D}\right)$ the informational value that can be assigned to the current transients $I_{s}\left(t\right)$. Using Equation (2) and transforming the conventional signal-to-noise ratio SN into informational language, three contributions to $i_{D}\left(E_{ph},T_{D}\right)$ can be identified:

(3) $$i_{D}\left(E_{ph},T_{D},V_{D},V_{b}\right)=i_{diss}\left(E_{ph},T_{D}\right)-i_{loc}\left(E_{ph},V_{gap}\right)-i_{time}(L,V_{b})$$

As discussed in our previous paper [25], the first contribution $i_{diss}\left(E_{ph},T_{D}\right)$ largely corresponds to the potential information $i_{pot}\left(E_{ph},T_{D}\right)$ that the travelling photon had carried prior to its detection. This first term simply measures the potential of a photon of energy $E_{ph}$ to generate entropy $S_{D}=E_{ph}/T_{D}$ inside a thermal reservoir maintained at a temperature $T_{D}$. In case this thermal reservoir is a detector operated at the temperature $T_{D}$, the generated information $i_{pot}\left(E_{ph},T_{D}\right)$ can only partly be retrieved as realized observational information $i_{D}\left(E_{ph},T_{D}\right)$:

(4) $$i_{D}\left(E_{ph},T_{D}\right)\leq i_{pot}\left(E_{ph},T_{D}\right)=\frac{1}{ln\left(2\right)}\frac{E_{ph}}{k_{B}T_{D}}.$$

The second and third contributions to $i_{D}$, which reduce $i_{D}$ below its maximum value of $i_{pot}$, arise when detector volumes are increased beyond their minimum sizes of $V_{D}={\left[\lambda /2\right]}^{3}$, where λ is the photon wavelength and when electron transit times $\tau_{t}$ are increased beyond the photon transit times $\tau_{ph}=L/c$ through the detector gap. Under these latter conditions externally captured photons are increasingly likely to coexist with internally generated black-body photons inside the gap, which can trigger photon detection events as well and which thereby reduce the confidence level of the produced photon-detection EOs. 

In the present paper we return to our previous work on PID detectors [25], and we focus on the special problem of single-photon detection and on those processes which turn single quantum-mechanical photon-detector interactions into macroscopically observable events, i.e., photon-detection EOs. Key result of these latter considerations is that the technical performance parameters of signal-to-noise ratio and/or their informational translations of $i_{D}\left(E_{ph},T_{D}\right)$ fall short of completely specifying the observational value of photon-detection EOs. The reason for this incomplete match is that signal-to-noise ratios and/or their informational translations do not specify the observational value of photon-detection EOs that had been gained by expanding the space-time volume of the initiating photon-detector interactions into the hugely enhanced spacetime dimensions of the ensuing photon-detection EOs. In order to amend this situation we derive in Section 4 and Section 5 two figures of merit (FOM), where the first FOM quantifies the level of macroscopic observability ${OV}_{EO}$ gained in a photon detection process, and the second the level of confidence ${SI}_{EO}$ that the observed photon-detection EO had actually been triggered by a photon that had originated from outside the detector itself. In Section 6 we discuss the entropy cost of observation. There, we show that EOs with optimum observability ${OV}_{EO}$ and optimum statistical significance ${SI}_{EO}$ can be obtained at minimum entropic cost in case photon and detector share evenly in the energetic cost of generating an EO. In Section 7 we consider the time evolution of EOs, and we show that, during their finite lifetimes, EOs proceed through the four stages of initiation, detection, erasure and reset. In this way a connection between PIDs and the widely discussed Szilard-type engines [27] and the Landauer principle [28,29,30,31,32] is established. In Section 8 we briefly summarize our results and present an outlook towards other types of EOs which are not photon-detection-related.

## 4. Making Microscopic Interaction Events Macroscopically Observable

Returning to Figure 3, we note that the triangular current pulses that emerge from PIDs in response to the internal absorption of a single photon can be described by [26]

(5) $$I_{s}\left(t,L,V_{b}\right)=2q\frac{t}{{\tau_{t}\left(L,V_{b}\right)}^{2}}\;;\;\left(0\leq t\leq \tau_{t}\right),$$

with

(6) $$\tau_{t}\left(L,V_{b}\right)=\frac{L}{c}\sqrt{\frac{2m_{e}c^{2}}{qV_{b}}}$$

standing for the transit time of the photoelectron through the electrode gap. In the two equations above, q stands for the electron charge, c for the speed of light and $m_{e}c^{2}$ for the rest energy of the photoelectron. An integration of $I_{s}\left(t,L,V_{b}\right)$ over the transit time interval $\left[0,\tau_{t}\right]$ yields the magnitude q of the transported charge, and thus establishes the fact of single-photon detection.

Multiplying Equation (5) by the bias potential $V_{b}$ that was applied across the electrode gap, the signal power emerges as

(7) $$P_{S}\left(t,L,V_{b}\right)=2qV_{b}\frac{t}{{\tau_{t}\left(L,V_{b}\right)}^{2}}.$$

A double integration of $P_{S}(t,L,V_{b})$ over the transit time interval $\left[0,\tau_{t}\right]$ first yields the kinetic energy $E_{kin}(L,V_{b})$ that the photoelectron had gained upon its impact at the anode surface,

(8) $$E_{S}(L,V_{b})=qV_{b},$$

and secondly the physical action that can be associated with the photoelectron transit and the concomitant production of an EO:

(9) $$W_{S}\left(L,V_{b}\right)=\frac{1}{3}qV_{b}\tau_{t}\left(L,V_{b}\right).$$

Considering that the function $W_{S}\left(L,V_{b}\right)$ has the dimension of physical action and that a travelling photon, prior to its detection, carried a single quantum of physical action h  towards the detector (Appendix A), a dimensionless measure of macroscopical observability of a detection event can be defined:

(10) $${OV}_{EO}(L,V_{b})=W_{S}\left(L,V_{b}\right)/h.$$

**Figure 4:**
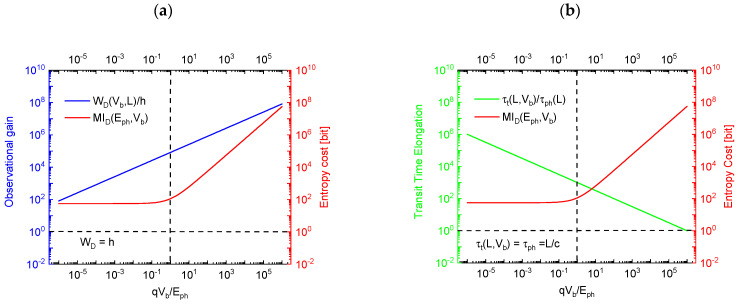
(**a**) Observational gain ${OV}_{EO}(L,V_{b})$ as measured in multiples of the Planck constant h versus the normalized bias potential $qV_{b}/E_{ph}.$ For the sake of good macroscopic observability, a device with a length extension of 300 μm≫λ was chosen $;\left(b\right)$ electron transit time through the electrode gap as a function of the normalized bias voltage. As shown in Section 6, the specific choices of $qV_{b}\approx E_{ph}$ represent conditions under which optimum observabilty is ensured at minimum entropic cost.

In Figure 4a, the variation of ${OV}_{EO}(L,V_{b})$ with increasing bias potential $V_{b}$ is shown. This first result shows that $W_{S}\left(L,V_{b}\right)$ can grow to large multiples of the Planck quantum of physical action h when the bias potential is increased. Photon detection EOs, therefore, can be regarded as hugely amplified images of the tiny packages of physical action that had been carried by the photon prior to its detection. Returning to Figure 4a, we additionally see that such gains in observability, ${OV}_{EO}(L,V_{b})$, need to be paid for in terms of entropy as the energetic photoelectrons will impact on the anode with increasingly larger energies as bias potentials are being increased and as this excess energy needs to be dissipated there. We mention here without proof that this entropy—when written in information units—amounts to

(11) $${MI}_{D}\left(E_{ph},V_{b},T_{d}\right)=\frac{1}{ln(2)}\left[\frac{E_{ph}+qV_{b}}{{k_{B}T}_{D}}\right].$$

We will come back to a discussion of Equation (11) in Section 6.

We have already shown in our previous paper that the figure of merit (FOM) of ${OV}_{EO}(L,V_{b})$ cannot be increased indefinitely, as the vacuum inside the electrode gap will break down and become permanently electrically conducing when the energy of the photoelectrons impacting on the anode increases beyond the threshold energy of electron–positron pair production of $E_{th}\geq {2m}_{e}c^{2}$.

Another possible option for arriving at even larger values of ${OV}_{EO}$ is increasing the electrode gap width L, or the detector volume $V_{D}=L^{3}$ as a whole. We will see in the following section that this latter option fails as huge detector volumes tend to contain large amounts of thermally generated radiation, i.e., noise photons, which deteriorate the statistical significance of the detected EOs. 

## 5. Statistical Significance of Detected Events

In addition to the observational value ${OV}_{EO}\left(L,V_{b}\right)$, which measures the level of spacetime expansion of an observed EO beyond the initiating photon-detector interaction event, the statistical significance ${SI}_{EO}\left(E_{ph},T_{D},L,V_{b}\right)$ of an observed EO can be defined as

(12) $${SI}_{EO}\left(E_{ph},T_{D},L,V_{b}\right)=1-\frac{1}{SN\left(E_{ph},T_{D},L,V_{b}\right)}$$

where $SN\left(E_{ph},T_{D},L,V_{b}\right)$ is the conventionally defined signal-to-noise ratio of a PID [26,33]. In this way another dimensionless FOM is obtained, which takes on the value of one when the observed EO has been generated by an outside photon, and the value of zero when the observed EO has been generated with equal probability by an outside or a thermally generated inside photon. In this first case of ${SI}_{EO}=1$, the photon detection EO conforms to the nature of an EO, as discussed in our previous paper [10]. There, we argued that EOs feature a double nature, being abstract pieces of binary information, on the one hand, and concrete physical entities endowed with the property of physical action, on the other hand.

With the signal-to-noise ratio SN standing for the ratio of the numbers of signal ($N_{s}$) versus noise ($N_{n}$) electrons inside the electrode gap, Equation (12) takes on a particularly simple form in the limit of single-photon detection, i.e., ($N_{s}=1$) and $N_{n}\approx 0$. In this latter case, the statistical significance of an EO reduces to the particularly simple form of

(13) $${SI}_{EO}\left(E_{ph},T_{D},L,V_{b}\right)=1-N_{n}.$$

Considering that the signal-to-noise ratio of PIDs is given by [25]

(14) $$SN\left(E_{ph},T_{D},L,V_{b}\right)=\frac{1}{\surd \pi }\sqrt{\frac{\left[\frac{E_{ph}}{k_{B}T_{D}}\right]exp\left[\frac{E_{ph}}{k_{B}T_{D}}\right]}{\left[\frac{V_{gap}}{V_{min}}\right]\left[\frac{V_{b\_max}}{V_{b}}\right]}},$$

it is revealed that the condition $N_{n}\approx 0$ is easily obtained by detecting high-energy photons in a detector operated at a temperature $T_{D}$ fulfilling the condition $E_{ph}\gg k_{B}T_{D}$. Or, in case this first condition cannot be fulfilled, another option is reducing the detector volume $V_{D}=L^{3}$ towards its minimum size of $V_{D\_min}={\left(\lambda /2\right)}^{3}$ with λ standing for the photon wavelength. Alternatively, the bias voltage of the PID can be raised towards its maximum value of $qV_{b\_max}$= $2m_{e}c^{2}$. Whereas reduced detector volumes are likely to run into conflict with the requirement of large levels of macroscopic observability ${OV}_{EO}$, the second measure drastically increases the entropy cost that needs to be paid for obtaining high levels of ${SI}_{EO}$ (see Figure 4b).

## 6. The Entropy Cost of Observation

As high levels of macroscopic observability and concomitantly high levels of statistical significance require contradictory demands on optimum detector volumes and biasing conditions, compromises need to be sought. A guiding principle towards optimizing both FOMs is considering the entropy costs that are associated with obtaining the particular values of ${OV}_{EO}$ and ${SI}_{EO}$ at the chosen level of bias potential $V_{b}$. 

**Figure 5:**
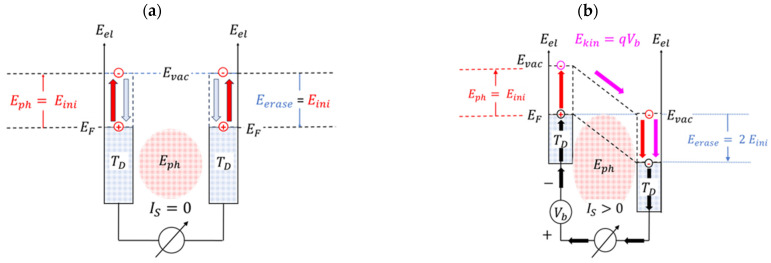
Pathways of photoelectrons in a band profile picture of a PID: (**a**) unbiased condition; (**b**) bias conditions optimally chosen to convert initiating photon energy into the kinetic energy of an emitted photoelectron (see Figure 6).

In order to assess the level of entropy production, consider Figure 5a,b. While Figure 5a shows, in the form of a semiconductor-like band profile, a PID as operated under zero-bias conditions, Figure 5b shows this same device with the applied bias potential satisfying the condition $qV_{b}=E_{ph}$.

Turning to Figure 5a first, it can be seen that with the electron work function $q\phi_{m}$ of the metal electrodes matching the photon energy $E_{ph}$ to be detected, a photon entering the detector can be absorbed at either of the two electrodes. Upon absorption in either of these electrodes, an electron may be raised from the Fermi energy $E_{F}$ of the affected electrode up to the vacuum level $E_{vac}$ at this same electrode. In such an event, the electron has gained potential energy amounting to $E_{pot}=E_{vac}-E_{F}=E_{ph}$. After having stayed at $E_{vac}$ for a very short time, the excited electron returns to the Fermi level and deposits its extra energy inside the electrode in the form of a small quantity of heat. With the electrode temperature having been raised above the ambient temperature $T_{D}$, the excess heat will subsequently flow away, either by means of heat conduction or by thermal radiation, thus re-establishing $T_{D}$ again. Further, as no electrical potential had been applied across the electrode gap, no displacement current had been induced, and no externally observable signal had been generated.

This situation drastically changes as a potential difference is applied between both electrodes. Once an electron has been excited to the vacuum level of the cathode, the excited electron does no longer return to the cathode Fermi energy but, rather, gets attracted by the electric field that was built up inside the electrode gap. With this field being present, the excited electron gets attracted to the vacuum level of the anode. Once it arrives there, the electron has gained a kinetic energy $E_{kin}=qV_{b}$ equivalent to the bias potential difference that had been applied across the electrode gap as shown in Figure 5b. With a current now flowing through the electrode gap, current continuity will assure that an electron current will not only be flowing through the electrode gap but also through the entire external circuit. As the current in this external circuit experiences friction, the energy $qV_{b}$ that is transported with this flow will finally be dissipated inside the external circuit and generate an entropy $S_{D1}=qV_{b}/T_{D}$ there. With the photoelectron having arrived at the vacuum level of the anode, the electron still has potential energy equivalent to the photon energy $E_{ph}$, as by assumption the electron work function $q\phi_{m}\;$ of both electrodes had satisfied the condition $q\phi_{m}=E_{ph}$. As in the case of Figure 5a, the collected electron will continue to fall onto the Fermi energy of the anode, thus producing another bit of entropy equivalent to $S_{D2}=E_{ph}/T_{D}$ there. Adding both entropic contributions and writing their sum in informational units [33,34], the result already reported in Equation 4 is retained. With ${MI}_{D}$ again being a dimensionless number, a logarithmic quantity $\Omega_{EO}$ can be defined that represents the gain in observational value relative to the entropy cost that had been invested at the particular level of bias potential:

(15) $$\Omega_{EO}\left(E_{ph},T_{D},L,V_{b}\right)=\frac{1}{ln(2)}ln\left\{\frac{1}{3}\left[\frac{k_{B}T_{D}}{h}\right]\left[\frac{\tau_{t}(L,V_{b})}{\left(1+\frac{E_{ph}}{qV_{b}}\right)}\right]\right\}.$$

In this latter equation

(16) $$\tau_{t\_red}\left(L,V_{b}\right)=\left[\frac{\tau_{t}(L,V_{b})}{\left(1+\frac{E_{ph}}{qV_{b}}\right)}\right]$$

is the reduced transit time of the electron through the electrode gap, and $\tau_{PB}\left(T_{D}\right)$ the Planck–Boltzmann thermalization time [32,35]

(17) $$\tau_{PB}\left(T_{D}\right)=\frac{h}{k_{B}T_{D}}.$$

Similarly, a second logarithmic function can be defined, which measures the statistical significance of the generated EO relative to its entropic cost:

(18) $$\Sigma_{EO}\left(E_{ph},T_{D},L,V_{b}\right)=\frac{1}{ln(2)}ln\left\{\frac{{SI}_{EO}\left(E_{ph},T_{D},L,V_{b}\right)}{{MI}_{D}\left(E_{ph},V_{b},T_{D}\right)}\right\}.$$

**Figure 6:**
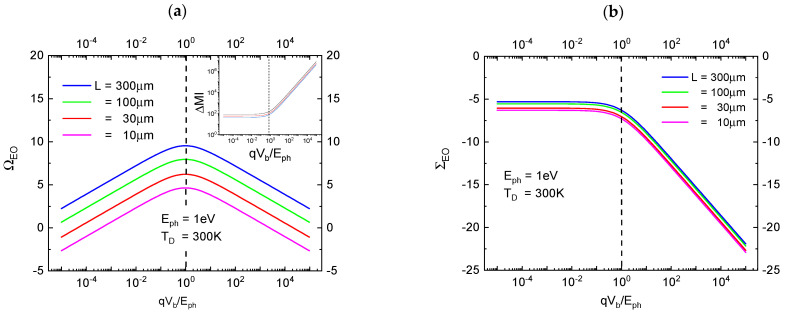
(**a**) Observability $\Omega_{EO}$ as a function of the normalized bias potential ${qV}_{b}/E_{ph}$ with the device size L as a parameter. The different curves in the inset show the impact of temperature on entropy production; (**b**) statistical significance $\Sigma_{EO}$ as a function of the normalized bias potential ${qV}_{b}/E_{ph}$ and as evaluated for different device sizes L. For clarity of presentation, the curves in (**b**) had been slightly offset from each other.

In Figure 6a,b, both FOMs are plotted as functions of the normalized bias potential $qV_{b}/E_{ph}$. Both figures show that an optimum compromise between high levels of macroscopic observability, statistical significance and minimum entropy cost can be obtained when the applied bias potential is chosen to match the energy of the travelling photon prior to its detection.

## 7. Time Evolution of Elementary Observations

**Figure 7:**
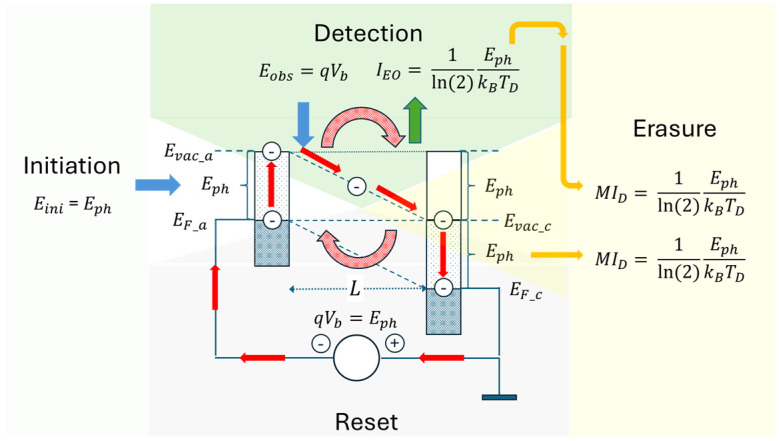
Time evolution of an EO as displayed in a band profile picture. Energy flows into the PID are denoted by blue arrows. Red arrows denote the cyclic progress of a photoelectron through the band profile during the four phases of initiation, detection, erasure, and reset. The green arrow indicates the outward flow of signal information when measured in conventional binary information units. Orange arrows denote outward entropy flows into the wider environments of the PID, causing erasure of the intermittently produced information. Reset to the pre-detection state is affected by the PID power supply, causing the electron to be lifted from the Fermi energy of the anode “upstairs” towards the Fermi energy of the cathode.

Accepting the above result of $qV_{b}=E_{ph}$, we now turn to the time evolution of an EO, i.e., to the kind of journey an excited photoelectron takes as it is circled through a PID device. This kind of travel is visualized in Figure 7, again in the form of a semiconductor-like band profile and with the electron travel directions being indicated by bold, red arrows.

In the first two steps of initiation and detection, external energy had to be introduced into the device in the forms of photon energy and externally supplied electrical energy; these inputs are indicated by bold, blue, inward-pointing arrows. With the excited electron moving through the electrode gap and the entire external circuit, a macroscopically observable electrical signal is generated at the sensor output during the detection phase. As this flow carries the energy $E_{EO}(L,V_{b})=qV_{b}$ (Equation (8)) that the electron had gained upon its travel through the electrode gap, a flow of potential information equivalent to

(19) $$i_{pot}\left(E_{ph},T_{D}\right)=\frac{1}{ln(2)}\frac{E_{ph}}{k_{B}T_{D}}$$

can be viewed as circling through the external circuit, and that is ready to transfer a maximum amount of information $i_{pot}$ to a potential observer. This flow of thermodynamic information is indicated by a bold, green and outward-pointing arrow on the detection side. Whether used for observational purposes or not, this potential information will ultimately end up as an increased amount of missing microscopic information inside the infinitely large thermal reservoir in which the detector had been embedded, and will thereby be erased:

(20) $${MI}_{env}\left(E_{ph},T_{D}\right)=\frac{1}{ln(2)}\frac{E_{ph}}{k_{B}T_{D}}$$

This latter effect of energy dispersion and entropy generation inside the reservoir is indicated by a thinner, wavy arrow pointing towards the right-hand side, and denoted by “Erasure”.

Up to this point, the photoelectron has progressed up to the vacuum level of the anode, where it still carries excess energy amounting to $E_{rest}=E_{ph}$. Upon returning to the anode Fermi energy, this energy will be dissipated inside the anode, thereby raising its temperature beyond the environmental temperature $T_{D}$, which will cause an outward energy flow into the reservoir, where a second piece of missing information will be created. In this way, the potential information of the electron, that had been sitting on the anode vacuum level, is finally erased. As in this downward transition no displacement current had been generated, the potential information in this latter case is directly erased, with no intermediate step of information gain.

As an overall result, one piece of potentially useful information, $i_{pot}$, had been generated during the detection phase, and two equally large pieces of missing information had been generated inside the thermal reservoir during the erasure phase. With the Landauer minimum amount of energy of $E_{La}=ln(2)k_{B}T_{D}$ per bit, the total energy expense for erasing one potentially useful bit of potential information is affected by the transfer of two units of energy of size $E_{La}$ to the thermal reservoir, thereby completely erasing the intermittently produced and potentially useful information that had been generated in the detection phase.

With the photoelectron having arrived at the anode Fermi energy, the photoelectron has lost all its acquired energy. In order to end up at a fully cyclic process of EO generation, information erasure, and reset, the photoelectron still needs to be “pumped up” by the external voltage source to arrive back at the cathode Fermi energy. In case this upward shift is not associated with any additional entropy production, the total energy expense in the initiation, detection, erasure and reset cycle still remains at two units of the Landauer minimum amount of energy, and larger amounts otherwise [31,32]:

(21) $$E_{erasure}\geq 2E_{La}=2\;ln\left(2\right)k_{B}T_{D}.$$

## 8. Summary and Conclusions

The results presented in this paper are a follow-up to our previous paper [10], in which we discussed historic experiments performed at the scale of atoms, nuclei and elementary particles, with an informational perspective in mind. There, it was shown that those experiments produced complex experimental answers that were composed out of streams of elementary observations (EOs) and which provide simple binary answers to the question of whether a matter–instrument interaction had taken place in the micro-domain of quantum phenomena or not.

**Figure 8:**
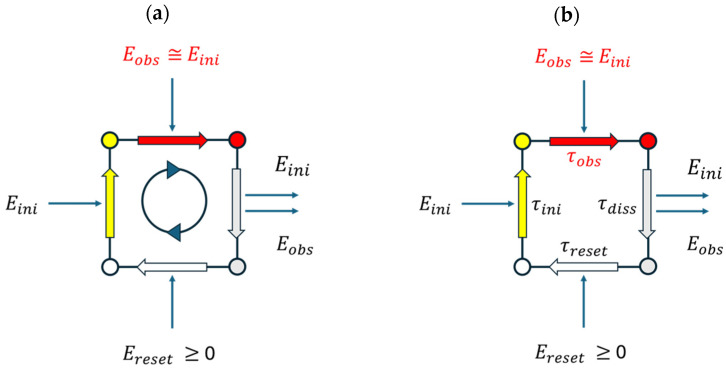
Generalized picture of EOs displayed as cyclic processes of initiation, detection, erasure and reset: (**a**) energy inputs and outputs in the different phases; (**b**) timing sequence of the four steps in response to energy inputs and outputs.

In the present paper, we have concentrated on the specific case of single-photon detection, and we have made use of the easily overseeable physics of PID photon detectors to develop a more detailed picture of EOs, both as novel physical entities and as pieces of abstract information. In brief, our key results are the following:

- EOs appear in the form of spatio-temporal transients with spatial dimensions larger than the observability limits set by the Abbe diffraction limit [36,37] and the temporal limits imposed by the Planck–Boltzmann equilibration time constant [32,35].
- Within the finite lifetime of EOs, EOs proceed through the four phases of initiation, detection, erasure and reset.
- EOs are pieces of physical action, formed at the expense of generating entropy and endowed with the informational properties of macroscopic observability $\Omega_{EO}$ and statistical significance $\Sigma_{EO}.$
- Once detected, EOs appear as macroscopic images of the initiating photon–detector interactions that had occurred at the micro-scale of quantum phenomena. The observability gain obtained in the micro–macro conversion of detection events can be measured in units of the Planck constant h. In the limit of $\Sigma_{EO}=1$, the generated EOs represent the binary answers concerning the initiating matter–instrument interactions that have already been discussed in our previous paper [10].
- The present investigations have further shown that EOs with optimum properties of $\Omega_{EO}$ and $\Sigma_{EO}$ are produced when photon and detector share evenly in the energetic and entropic costs required for turning unobservable micro-events into macroscopically observable EOs. This picture of EO formation is in accordance with the view of a participatory process of information gain [9].
- Once the detection phase of EOs has ended, both the energy of the initiating photon and the energy supplied by detector-internal resources are dissipated and turned into missing information $\Delta MI_{env}$ concerning the unobservable microstate of the wider environment of the PID.
- After energy dissipation and spatial dispersion have taken place, the intermittently generated information has been removed from the PID device and has been distributed in the wider environment of the PID and, thus, been erased. In terms of energy consumption, this erasure has been performed at the expense of transferring two units of the Landauer minimum energy bound of $E_{La}={ln\left(2\right)k}_{B}T_{D}$ per bit from the PID and towards the thermal reservoir in which the PID had been embedded. In the final step of reset, additional energy needs to be supplied from external resources to reset the instrument for a new round of photon detection. In cases where this final step is associated with an additional entropy production, the total energy cost for erasure and reset exceeds the Landauer minimum energy cost of two units of $E_{La}.$
  (22) $$E_{erasure}\geq 2E_{La}=2\;ln\left(2\right)k_{B}T_{D}.$$
- Looking beyond the field of photon detection, we propose that the above considerations regarding photon detection may be generalized in diagrams, as displayed in Figure 8. In this figure, the cyclic process of EO initiation, detection, erasure and reset is displayed in two diagrams, with the first one emphasizing the energy inputs and outputs in the course of an EO cycle, and the second focusing on the timing issues in response to the energy inputs and outputs.

Assuming that $E_{ini}\gg k_{B}T$, the parameter $\Omega_{EO}$ is the dominant figure of merit (FOM), which characterizes the observational value of an EO. Following Equation (15), this can be approximated as

(23) $$\Omega_{EO}(E_{ph},T_{D},L)≅\frac{1}{ln(2)}ln\left[\frac{\tau_{obs}}{\tau_{PB}(T_{D})}\right].$$

High values of $\Omega_{EO}$ obviously rely on the efficiency of extending the observational time span $\tau_{obs}$ of EOs beyond the Planck–Boltzmann equilibration time of $\tau_{PB}\left(T_{D}\right)=h/k_{B}T$, which is a combination of the two natural constants of h and $k_{B}$.

Turning to the parameter $\tau_{obs}$, the comparison in Table 1 shows that $\tau_{obs}\;$ is most likely that EO-related parameter that exhibits the largest range of variability. This latter comparison shows that EOs should not be confused with the reversible thermal fluctuations of size $E_{ini}\gg k_{B}T_{D}$ that can occur within a heat reservoir of temperature $T_{D}$, and whose lifetimes $\tau_{H}$ are dictated by the Heisenberg time–energy uncertainty relationship, and that, in the case of large energies $E_{ini}$, are much shorter than the Planck–Boltzmann thermalization time of $\tau_{PB}(T)$ [32,35]:

(24) τH=hEini≅10−15s≪τPBTD=hkBTD≅10−13s.

Extremely long times of $\tau_{obs}$, as for instance in photography, rather point to the fact that the art of creating particularly long-lived EOs relies on the art of using the energy inputs $E_{ini}$ and $E_{obs}$ to drive the detection instrument into a deeply trapped observational state with huge thermal release times. Principally, such a situation of deep trapping can be achieved by large energy barriers of $E_{reset}$ and long associated waiting times $\tau_{reset}$, which both inhibit a detector reset and which trap the detector in a long-lived observational state.

Overall, what we have achieved in the end is the introduction of a new vehicle of experimental information gain and information erasure that goes beyond the traditional Szilard cylinder and piston-type approaches [27], which had been borrowed from the age of steam engines. The proposed picture of EOs is much closer to actual experiments [10] that had been performed in unravelling processes inside the quantum domain. EOs, in addition, involve interactions of single particles with detection instruments and, thus, more directly conform to the requirements of minimum thermal engines.

## Figures and Tables

**Table 1:** Observational lifetimes and EO figures of merit in different experimental circumstances.

EO Origin	Thermal Fluctuation Time $\tau_{PB}(RT)$	Observational Lifetime $\tau_{obs}$	FOM_EO_
Photon detection (PID) This work	$1.5\times 10^{-13}s$	$≅10^{-9}s$	≅12
α-particle detection(fluorescence) [12,13]	$1.5\times 10^{-13}s$	$≅10^{-8}s$	≅17
Wilson cloud chamber [18]	$1.5\times 10^{-13}s$	≅3s	≅44
Double-slit experiments (photography) [14]	$1.5\times 10^{-13}s$	$≅100\;a≅3\times 10^{9}s$	≅74

**Figure A1:**
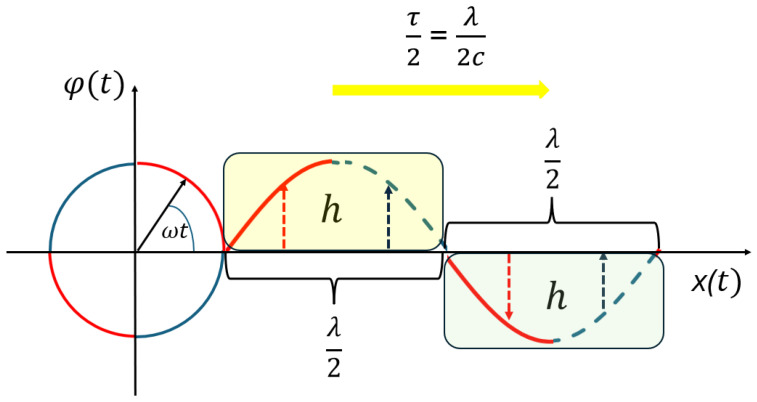
Propagation of an electromagnetic wave φ(x,t) as visualized as a shift of packages of physical action of the size of a single Planck unit h.

## Data Availability

All relevant data are contained in the main text.

## Appendix A. Photon Propagation

In the main text, we have arrived at the conclusion that EOs are physical entities endowed with the property of physical action $W_{EO}\left(L,V_{b}\right)\gg h.$ In order to support our interpretation that EOs can be regarded as hugely amplified images of the tiny packages of physical action h that had been carried with the photons prior to their detection, we show in this appendix that freely propagating electromagnetic waves can be viewed as repeatedly generating and erasing single quanta of physical action h.

With φ(x,t) standing for one of the electrical or magnetic field components, plane electromagnetic waves can be described by

(A1) $$\varphi (x,t)=\sin\left\{2\pi \left[\frac{x}{\lambda }-\frac{t}{\tau }\right]\right\}$$

where λ stands for the photon wavelength and τ for the photon vibrational period. Physically, such sine wave functions arise from the fact that within each half-wave period, time-varying electric fields generate time-varying magnetic fields, and vice-versa. As predicted by Maxwell’s equations, such electromagnetic field disturbances move forward along the x-axis with a phase velocity of c=νλ until the wave either becomes absorbed in a solid piece of matter or inside a detector, where the field disturbance can trigger a detection event.

Mathematically, sine waves, as described above, can be constructed by rotating a unit vector around the origin of a circle with a unit radius and with its terminal point moving along its periphery with angular speed ω=2π/τ and spanning an arc length ranging from 0 to 2π (Figure A1). The sine wave in Figure A1 can also be viewed in a somewhat different manner by replacing λ and τ=1/ν with their quantum analogs of $\lambda =h/p_{ph}$ and $\tau =h/E_{ph}$, where $E_{ph}$ and $p_{ph}$ stand for the photon energy and the photon momentum and h for Planck’s constant. With these substitutions, Equation (A1) takes the form

(A2) $$\varphi (x,t)=\sin\left\{2\pi \left[\frac{p_{ph}x-E_{ph}t}{h}\right]\right\}.$$

This latter form can be interpreted in the way that the repeated interconversion of electric into magnetic fields and vice versa gives rise to a repeated generation and erasure of quanta of physical action of size h, which causes these quanta to be shifted along the x-axis in discrete steps of length Δx = λ/2. In Figure A1, this kind of transport is indicated in the form of colored boxes. Specifically, these boxes indicate that within the first quarter of each full wave, a quantum of physical action is gradually generated, whereas it is gradually erased as it moves through the second quarter-wave period. Upon entering the second half-wave period, the package is re-generated and re-erased again, and then arrives with zero amplitude at the onset of the second full-wave period again. In essence, this view of wave propagation can also be viewed as an alternative form of particle motion in which the quanta of physical action are shifted in discrete steps whenever an interconversion of electrical into magnetic fields and vice versa occurs within the driving electromagnetic wave that propagates into the x-direction.

As such a kind of transport cannot be ascertained by direct observation, we call these discrete shifts of action quanta “propagational events”. In the main text, we are concerned with the transformation of “propagational events” into macroscopically observable “photon detection EOs” with the help of a conceptual device. These latter considerations confirm the point of view that photon detection EOs are hugely amplified images of the initiating quanta of physical action of size h.
